# Teilhabe von Menschen mit Behinderung am Arbeitsmarkt: Förderfaktoren und Barrieren aus rechtlicher Sicht

**DOI:** 10.1007/s00103-026-04267-x

**Published:** 2026-07-13

**Authors:** Felix Welti

**Affiliations:** Institut für Sozialwesen, Universität Kassel, Arnold-Bode-Straße 10, 34109 Kassel, Deutschland

**Keywords:** Angemessene Vorkehrungen, Arbeit, Erwerbsminderung, Recht, Rehabilitation, Invalidity, Reasonable accommodation, Rehabilitation, Rights, Work

## Abstract

Die Teilhabe von Menschen mit Behinderungen am Arbeitsleben ist eine wesentliche Dimension gesellschaftlicher Teilhabe. Die UN-Behindertenrechtskonvention, das EU-Recht und das Grundgesetz verpflichten dazu, das gleiche Recht von Menschen mit Behinderungen auf Arbeit zu sichern. Rechtlich verankert sind insbesondere Beschäftigungspflicht und Ausgleichsabgabe, Diskriminierungsverbote und angemessene Vorkehrungen, Barrierefreiheit von Arbeitsplätzen sowie die Interessenvertretung durch die Schwerbehindertenvertretung. Strittig ist die Rolle geschützter Beschäftigung. Zudem bestehen Wechselwirkungen der sozialen Sicherung bei Erwerbsminderung mit den Rechten auf Bildung, Gesundheit und Rehabilitation. Insgesamt besteht hoher Forschungsbedarf.

## Abstract (en)

Participation of disabled persons in working life is a key dimension of participation in society. The UN Convention on the Rights of Persons with Disabilities, European Union Law and the German Basic Law all mandate equal rights of disabled persons to work. For this reason, occupation duty and compensational levy, discrimination bans and duties to reasonable accommodation, and the accessibility of working places exist. The role of sheltered work is controversial. There are interactions between social security in cases of invalidity and the realization of the rights to education, health and rehabilitation. Further research is urgently needed.

## Einleitung

Staat, Gesellschaft und soziale Sicherung bauen auf Arbeit auf, heute überwiegend durch den Arbeitsmarkt vermittelte abhängige Erwerbsarbeit. Arbeit ist auch ein wesentlicher Faktor von individueller Würde [1], Identität und kollektiver Einbeziehung. Behinderung als Phänomen und Rechtsbegriff [2] ist insofern auf Arbeit bezogen. Gesellschaftliche Teilhabe wird zu einem wichtigen Teil durch Arbeit vermittelt. Ein sozialer Rechtsstaat steht in der Verantwortung, Menschen mit Behinderungen Teilhabe an Arbeit zu ermöglichen. Dabei ist zu beachten, inwieweit sich rechtliche Regelungen als fördernde Faktoren oder als Barrieren der Teilhabe am Arbeitsleben erweisen. Das Rechtssystem kann an der Schnittstelle zur Politik und in gerichtlich ausgetragenen Konflikten Ziele und Zwecke der Regelungen bestimmen. Das für die Beurteilung ihrer Geeignetheit und Angemessenheit erforderliche empirische Wissen kann es jedoch nicht selbst bereitstellen. Hierzu wird der Austausch mit den Wissenschaften benötigt, die sich mit Gesundheit, Arbeitsmarkt und Teilhabe von Menschen mit Behinderungen befassen [3]. Diese sollten ihrerseits über die rechtlichen Regelungen und ihre möglichen Wirkweisen hinreichend informiert sein. Dokumentationspflichten und Statistiken unterstützen sie dabei.

## UN-Behindertenrechtskonvention

Die UN-Behindertenrechtskonvention (UN-BRK) ist durch ihre Ratifikation seit 2009 Teil der deutschen Rechtsordnung und beeinflusst die Auslegung und Entwicklung des Verfassungsrechts, des Arbeitsrechts und des Sozialrechts [4]. Sie beinhaltet in Art. 27 UN-BRK das „gleiche Recht auf Arbeit“ [5]. Dies ist als soziales Menschenrecht nach und nach zu verwirklichen [6]. Der Staat darf nicht untätig bleiben, sondern muss die Bedingungen eines inklusiven Arbeitsmarkts und zugänglicher Arbeitsplätze schaffen. Den Kern bildet das Verbot, Menschen mit Behinderungen zu diskriminieren. Es umfasst auch ein Gebot zu aktivem Tun, zu angemessenen Vorkehrungen [7]. Die UN-BRK hat mittlerweile 186 Vertragsstaaten, fast alle, außer den USA. Sie beeinflusst die Auslegung der Menschenrechtsverträge, auch der Europäischen Menschenrechtskonvention (EMRK). Durch die regelmäßigen Staatenberichtsverfahren [8] institutionalisiert sie auch Vergleich und Kritik am Stand ihrer Umsetzung und regt Politik und Wissenschaft an, deren Voraussetzungen zu reflektieren [9]. Das Potenzial der Berichterstattung zur UN-BRK für die weltweit vergleichende Forschung wird bislang nicht ausgeschöpft.

## EU-Recht

Das Diskriminierungsverbot wegen Behinderung im Arbeitsleben, einschließlich des an die Arbeitgeber gerichteten Gebots angemessener Vorkehrungen, wurde erstmals 2000 durch die Gleichbehandlungsrahmenrichtlinie 2000/78/EG für die Europäische Union festgeschrieben. Es hat seitdem Eingang in die Rechtsprechung des Europäischen Gerichtshofs und der Arbeitsgerichte gefunden. Seit 2010 ist die EU der UN-BRK beigetreten, sodass sich die Normen wechselseitig beeinflussen [10]. Durch regelmäßige Berichte und Diskussionen trägt die EU zum Diskurs bei [11]. Dies könnte noch stärker für die nationalstaatlich bezogene Arbeitsmarkt- und Gesundheitsforschung genutzt werden.

## Deutsches Verfassungsrecht

Mit der sozialstaatlichen Aufgabe, die Teilhabe von Menschen mit Behinderungen am Arbeitsleben zu sichern, werden schon lange Instrumente wie die Beschäftigungspflicht und Ausgleichsabgabe als gerechtfertigte Eingriffe in die Freiheit der Arbeitgeber angesehen [12]. Dass sie auch unmittelbar Adressaten des Diskriminierungsverbots sind, hat sich erst durch das EU-Recht und die UN-BRK durchgesetzt [13]. Das 1994 eingeführte Benachteiligungsverbot in Art. 3 Abs. 3 Satz 2 Grundgesetz (GG) wird in diesem Licht auch auf den staatlichen Rahmen des Arbeitsmarktes bezogen. Die Folgen dieser gesteigerten Verantwortung zu konkretisieren, ist zunächst eine rechtsdogmatische Aufgabe. Je stärker der Staat Arbeitgeber und Unternehmen in die Pflicht nimmt – und ebenso, wenn er dies bewusst unterlässt –, desto mehr sind Erkenntnisse über die Wirkungen rechtlicher Regelungen erforderlich. Nur so sind rechtliche Abwägungen nicht allein zwischen guten Absichten, sondern auch zwischen deren Folgen möglich [14].

## Beschäftigungspflicht

Die Pflicht, schwerbehinderte Menschen zu beschäftigen, ist seit mehr als 100 Jahren Teil der deutschen Rechtsordnung [15]. Sie entstand aus der Not nach dem Ersten Weltkrieg, bezogen auf Kriegsbeschädigte, und ist seit 1974 verallgemeinert. Betriebe mit mindestens 20 Beschäftigten müssen heute 5 % schwerbehinderte Menschen beschäftigen (§ 154 Abs. 1 Satz 1 SGB IX), wobei besonders schwer behinderte Beschäftigte und Auszubildende mehrfach zählen (§ 159 SGB IX). Tun sie dies nicht, müssen sie eine Ausgleichsabgabe zahlen, die nach dem Grad der Erfüllung progressiv gestaffelt ist (§ 160 SGB IX) und zuletzt für diejenigen Betriebe erhöht worden ist, die gar keine schwerbehinderten Menschen beschäftigen. Die Ausgleichsabgabe ist keine Strafe, sondern hat die Funktion, das wirtschaftliche Handeln zu lenken und einen Ausgleich herzustellen, da sie zweckgebunden zur Förderung der Beschäftigung schwerbehinderter Menschen verwendet wird. Insbesondere werden die begleitenden Hilfen im Arbeitsleben der Integrationsämter für Beschäftigte und Arbeitgeber (§ 185 SGB IX) finanziert. Die vorsätzliche Verletzung der Beschäftigungspflicht ist keine zusätzlich strafbewehrte Ordnungswidrigkeit mehr. Sie kann aber ein Indiz für verbotene Diskriminierung sein [16]. Da die Beschäftigungspflicht und Ausgleichsabgabe an den Status anknüpfen, als schwerbehindert anerkannt zu sein, sollten das Anerkennungsverfahren und die ihm zugrunde liegende Versorgungsmedizinverordnung (§§ 153, 153a SGB IX) wirklichkeitsnah und orientiert an der modernen Systematik der ICF (International Classification of Functioning, Disability and Health) typische Beeinträchtigungen der Teilhabe durch Gesundheits- und Funktionsstörungen abbilden [17]. Beschäftigungspflicht und Ausgleichsabgabe sind Instrumente, die schon lange in vielen Staaten genutzt werden [18]. Vergleichende Forschung über die Bedingungen der Wirksamkeit auf der betrieblichen Ebene und im Arbeitsmarkt als Ganzen wäre wünschenswert.

## Diskriminierungsschutz und angemessene Vorkehrungen

Im Allgemeinen Gleichbehandlungsgesetz (§§ 6–8 AGG) und im Schwerbehindertenrecht (§ 164 Abs. 2 SGB IX) ist geregelt, dass Menschen wegen ihrer Behinderung beim Zugang zu Arbeitsverhältnissen, bei ihrer Beendigung und ihrer Ausgestaltung nicht benachteiligt werden dürfen. Da benachteiligende Absichten in einem Einstellungs- oder einem Kündigungsverfahren oft schwer zu objektivieren sind, konkretisiert sich das Diskriminierungsverbot auch in Verfahrenspflichten, etwa der Einbeziehung der Schwerbehindertenvertretung (§ 178 Abs. 2 SGB IX), und der Identifikation mittelbar benachteiligender Kriterien und Rahmenbedingungen.

Diskriminierung ist nicht nur durch benachteiligendes Tun möglich, sondern auch durch das Unterlassen angemessener Vorkehrungen (Art. 5 Abs. 3 und Art. 2 UN-BRK; § 7 Abs. 2 Behindertengleichstellungsgesetz, BGG), mit denen gleiche Rechte und Chancen im Einzelfall hergestellt werden können [19]. Solche Anpassungen von Arbeitsbedingungen, Arbeitszeiten oder Arbeitsplätzen sind für schwerbehinderte Menschen gesetzlich vorgeschrieben (§ 164 Abs. 4 SGB IX). Ob sie auch im Übrigen aus der richtigen Auslegung der arbeitsrechtlichen Generalklauseln (insbesondere § 618 BGB, § 106 GewO) folgen, ist im Einzelnen Gegenstand von Rechtsprechung und Wissenschaft [20]. Verbreitet wird die Klarstellung gewünscht, dass angemessene Vorkehrungen im Arbeitsverhältnis grundsätzlich geboten sind [21]. Die Angemessenheit für Arbeitgeber richtet sich auch danach, ob Vorkehrungen durch Rehabilitationsträger oder das Integrationsamt unterstützt werden (Art. 5 RL 2000/78/EG), wozu rechtlich regulierte Suchprozesse wie das Betriebliche Eingliederungsmanagement (§ 167 SGB IX) beitragen können. Umgekehrt kann die Wirksamkeit sozialrechtlicher Regulierungen erst im Zusammenhang mit ihrer Akzeptanz und Nutzung in den Unternehmen beurteilt werden.

## Barrierefreiheit

Art. 27 UN-BRK spricht die Zugänglichkeit (Art. 9 UN-BRK) von Arbeitsplätzen an. Nach der Arbeitsstättenverordnung (§ 3 ArbStV) ist Barrierefreiheit nur geboten, wenn Menschen mit Behinderungen beschäftigt werden. Diese Regelung ist problematisch, weil sie Benachteiligungen bei der Einstellung fördern kann, wenn Arbeitgeber meinen, ohne behinderte Beschäftigte nicht verpflichtet zu sein. Zudem werden Barrierefreiheit und universelles Design als Gestaltungsprinzipien besser gefördert, wenn sie von vornherein bei Planungsprozessen für eine unbestimmte Anzahl von Nutzerinnen und Nutzern von Gebäuden und gestalteten Umwelten zugrunde gelegt werden. Entsprechend fordern die Bauordnungen der Länder und die Behindertengleichstellungsgesetze von Bund und Ländern sowie das allgemeine Sozialrecht (§ 17 Abs. 1 Nr. 4 SGB I) für eine Vielzahl öffentlich zugänglicher Gebäude und Informationstechniken Barrierefreiheit [22]. Ergänzt wird dies aus Sicht der Verbraucherinnen und Verbraucher durch das zivilrechtliche Barrierefreiheitsstärkungsgesetz (BFSG; [23]). Der Stand der Barrierefreiheit von öffentlichen Gebäuden und Arbeitsstätten ist tiefergehend kaum erforscht, ebenso die konkrete Wirkung von Barrieren. Ein stärkerer Forschungsstand würde es ermöglichen, bei den notwendigen längerfristigen Planungen zur Herstellung von Barrierefreiheit diejenigen Maßnahmen zu priorisieren, mit denen Teilhabe am besten gefördert werden könnte. Zudem würden Erkenntnisse über die typischerweise in Arbeitswelt und Gesellschaft erreichte Barrierefreiheit bei der Entscheidung über die Maßstäbe des Schwerbehindertenrechts helfen, die Beeinträchtigung der Teilhabe realistisch einzuschätzen.

## Geschützte oder unterstützte Beschäftigung?

Zu den umstrittensten Fragen der Teilhabe von Menschen mit Behinderungen am Arbeitsleben gehört die geschützte Beschäftigung. In vielen Ländern bestehen öffentlich geförderte Einrichtungen, in denen Menschen, meist mit kognitiven und psychischen, teils auch schweren körperlichen Einschränkungen unter besonderen Bedingungen produktive Arbeit leisten. Diese Einrichtungen der geschützten Beschäftigung werden vom Ausschuss der Vereinten Nationen für die Rechte von Menschen mit Behinderungen in seinen Allgemeinen Bemerkungen zum Recht auf Arbeit und in den Abschließenden Bemerkungen zu zahlreichen Staatenberichten kritisiert; zugleich werden Strategien für einen Ausstieg aus der geschützten Beschäftigung in gesonderten Einrichtungen zugunsten einer unterstützten Beschäftigung auf dem allgemeinen Arbeitsmarkt gefordert [24].

In Deutschland bestehen die Werkstätten für behinderte Menschen (WfbM; § 219 SGB IX), die seit 1974 mit einer Rechtsgrundlage im Schwerbehindertenrecht tätig sind und in denen aktuell mehr als 300.000 Personen arbeiten. Sie bestehen aus einem Berufsbildungsbereich, in dem regelhaft die Leistungen von der Bundesagentur für Arbeit finanziert werden (§ 57 SGB IX), und einem Arbeitsbereich, in dem meist die Träger der Eingliederungshilfe für die Leistungen verantwortlich sind (§ 58 SGB IX). Nach ihrem gesetzlichen Auftrag sind sie zugleich Arbeitsplätze und Rehabilitationseinrichtungen mit dem Ziel einer Beschäftigung auf dem allgemeinen Arbeitsmarkt. Dies wird jedoch nur selten erreicht. Für die Beschäftigten der WfbM gelten im Rahmen eines arbeitnehmerähnlichen Rechtsverhältnisses (§ 221 SGB IX) zahlreiche Regelungen des Arbeitsrechts und der Zugang zu Kranken‑, Renten- und Unfallversicherung (§ 5 Abs. 1 Nr. 7 SGB V; § 1 Satz 1 Nr. 2 SGB VI; § 2 Abs. 1 Nr. 4 SGB VII), nicht jedoch der Mindestlohn und die Arbeitslosenversicherung. Das Entgelt liegt einschließlich Arbeitsförderungsgeld (§ 59 SGB IX) deutlich unter dem Mindestlohn (§ 221 Abs. 2 SGB IX) und wird mit Grundsicherung (§ 41 SGB XII) oder Erwerbsminderungsrente (§ 43 SGB VI) aufgestockt. Zugangsvoraussetzung zur WfbM ist, dass eine Beschäftigung auf dem allgemeinen Arbeitsmarkt aktuell nicht infrage kommt, aber ein Mindestmaß an wirtschaftlich verwertbarer Arbeitsleistung erbracht wird (§ 219 Abs. 2 SGB IX). Dieser Personenkreis der WfbM kann auch mit einem Budget für Arbeit auf dem allgemeinen Arbeitsmarkt arbeiten (§ 61 SGB IX). Dies besteht in einem Lohnkostenzuschuss und individuell benötigter Unterstützung. Bislang wird das Budget für Arbeit nur von wenigen Menschen genutzt.

Der faktisch generelle Ausschluss des Mindestlohns in WfbM kann als diskriminierende Praxis oder Rechtslage interpretiert werden [25]. Die Einkommenssituation und die Übergangschancen von WfbM-Beschäftigten zu verbessern, ist ein häufig genanntes politisches Ziel. Ob der Weg dazu in einer generellen Annäherung an den allgemeinen Arbeitsmarkt bestehen sollte, ist jedoch strittig. Diskutiert wird auch, den Berufsbildungsbereich stärker vom Arbeitsbereich zu trennen, um den regelhaften Übergang in den Arbeitsbereich, oft mit lebenslanger Beschäftigung, stärker infrage zu stellen [26]. Auch der weitgehende Ausschluss der Bundesagentur und der Rentenversicherung aus der Verantwortung für die Rehabilitation Werkstattbeschäftigter ist ein Problem [27]. Insgesamt besteht noch erheblicher Forschungsbedarf, auch im internationalen Vergleich der geschützten Beschäftigung und der Alternativen zu ihr.

## Erwerbsminderung und soziale Sicherung

Zu den Folgen beeinträchtigter Teilhabe an Erwerbsarbeit gehört, dass die Fähigkeit, Erwerbseinkommen zu erzielen, gemindert ist. Dieses soziale Risiko stand am Anfang der Entwicklung der deutschen Sozialversicherung [28]. Heute wird es vor allem durch die Erwerbsminderungsrenten der Rentenversicherung und die Grundsicherung der Sozialhilfe gesichert. Definiert ist die volle Erwerbsminderung durch die Fähigkeit, weniger als 3 Stunden am Tag unter den Bedingungen des allgemeinen Arbeitsmarkts erwerbstätig sein zu können (§ 43 Abs. 2 SGB VI). Mit dieser medizinisch und arbeitsmarktbezogen schwer zu objektivierenden Feststellung ist heute auch die Zuständigkeitsgrenze zwischen Jobcenter und Sozialamt bei existenzsichernden Leistungen (§ 8 Abs. 1 SGB II) und, mit Blick auf die Erreichbarkeit der Erwerbsfähigkeit als Ziel, bei der medizinischen Rehabilitation zwischen Rentenversicherung und Krankenkasse verbunden. Damit kann sich der Ausschluss vom allgemeinen Arbeitsmarkt und seinen Unterstützungssystemen verfestigen. In Verbindung mit Zuverdienstgrenzen (§ 96a SGB VI) führt das zu einer Trennung zwischen Personen, die dem Arbeitsmarkt, und solchen, die dem Ausschluss vom Arbeitsmarkt zugeordnet sind. Das eigentliche Ziel der Feststellung von Erwerbsminderung, geringere Einkommenschancen auszugleichen, rückt in den Hintergrund [29]. Insoweit besteht Forschungsbedarf, ob andere Sozialsysteme, etwa in Österreich, den Niederlanden oder Finnland [30], diese Schnittstelle besser bearbeiten und wie die Systeme der Rückkehr zur Arbeit weltweit funktionieren [31].

## Bildung und Arbeit

Art. 24 UN-BRK schreibt das Recht auf Bildung für Menschen mit Behinderungen fest [32]. Benachteiligungen im Zugang zu Bildung schreiben sich im Zugang zu Erwerbsarbeit fest. Exklusive Bildungseinrichtungen wie Förderschulen finden oft ihre Fortsetzung in dem alternativlos scheinenden Weg in geschützte Beschäftigung [33]. Auch Übergänge von Menschen mit Behinderungen zur Hochschule und von der Hochschule in den Arbeitsmarkt sind erschwert [34]. Diese Zusammenhänge sind im Einzelnen noch näher aufzuklären.

## Gesundheit und Arbeit

Das in Art. 25 UN-BRK festgeschriebene Recht auf bestmögliche Gesundheit für Menschen mit Behinderungen dient insbesondere dem gleichberechtigten Zugang zu Leistungen und Einrichtungen des Gesundheitssystems. Dieser ist, insbesondere wegen fehlender Barrierefreiheit der Einrichtungen und angemessener Vorkehrungen durch sie und die Beschäftigten des Gesundheitssystems, nicht immer gegeben [35], obwohl geboten [36]. Auch hierdurch kann die Teilhabe an Arbeit beeinträchtigt werden.

## Rehabilitation und Arbeit

Art. 26 UN-BRK weist Rehabilitation als Teil der Maßnahmen aus, zu denen die Vertragsstaaten verpflichtet sind und die auch der Realisierung des Rechts auf Arbeit dienen. Rehabilitation wird dabei als multidisziplinär ausgewiesen und schließt Unterstützung durch andere Menschen mit Behinderungen („peer support“) ein. Im deutschen Rehabilitationsrecht ist der enge Bezug zur Arbeit durch die Festschreibung der stufenweisen Wiedereingliederung (§ 44 SGB IX) in den Betrieb und das Betriebliche Eingliederungsmanagement unter Mitwirkung der Rehabilitationsträger (§ 167 SGB IX) ausgewiesen [37]. Die Förderung der Selbsthilfegruppen und Selbsthilfeorganisationen (§ 45 SGB IX) sowie der Ergänzenden Unabhängigen Teilhabeberatung (§ 32 SGB IX) zeigen, dass auch Unterstützung durch Gleichbetroffene Teil der Rehabilitation ist. Zur Wirksamkeit der Rehabilitation im Ganzen wie ihrer genannten Teile besteht erheblicher Forschungsbedarf [38].

## Data Availability

Alle dieser Arbeit zugrunde liegenden Daten sind in diesem Artikel enthalten.
